# Supplementary material for: EnzML: multi-label prediction of enzyme classes using InterPro signatures
Source: BMC Bioinformatics. 2012 Apr 25;13:61. doi: 10.1186/1471-2105-13-61 (PMC3483700; doi:10.1186/1471-2105-13-61)
Supplement: Addtional file 5 — The Java code to format the data files, evaluate and predict. The file enzml_java_code.tar.gz contains the Java code used to format database data to ARFF and XML formats, to execute cross and train-test (jackknife) evaluations and to record evaluation results to database. More information is included in the readme.txt file and the Javadoc files. The code can be used with a MySQL database. To use a different database software, other JDBC drivers might be required. [file 1471-2105-13-61-S5.gz › java_code/enzml2011/doc/uk/ac/ed/inf/csb/taweka/ArffFileTableWriter.html]

ArffFileTableWriter


---


|  |  |  |
| --- | --- | --- |
| **Package** \| **Class** \| **Use** \| **Tree** \| **Deprecated** \| **Index** \| **Help** | |  |
| PREV CLASS   **NEXT CLASS** | **FRAMES**    **NO FRAMES**     **All Classes** |  |
| SUMMARY: NESTED \| FIELD \| CONSTR \| METHOD | DETAIL: FIELD \| CONSTR \| METHOD |  |


---


## uk.ac.ed.inf.csb.taweka Class ArffFileTableWriter

```
java.lang.Object
  uk.ac.ed.inf.csb.taweka.ArffFileTableWriter
```

---

``` public class ArffFileTableWriter extends java.lang.Object ```

An ArffFileTableWriter object writes the database table
that contains the elements essential to build an arff file
(the name of the arff file, the query to select instances, attributes, class values etc)

**Version:**
:   16-Apr-2007

**Author:**
:   Luna

---

**Constructor Summary**

`ArffFileTableWriter(uk.ac.ed.inf.csb.utils.databaseutils.DbConnProps dbConn)`
  
          Constructor 1: initialises the table (uses given database connection file)


| **Method Summary** | |
| --- | --- |
| `void` | `createArffFileTable()`             Creates the table for the ARFF file records |
| `static void` | `main(java.lang.String[] args)`             Main for testing |

**Methods inherited from class java.lang.Object**

`equals, getClass, hashCode, notify, notifyAll, toString, wait, wait, wait`

**Constructor Detail**

### ArffFileTableWriter

```
public ArffFileTableWriter(uk.ac.ed.inf.csb.utils.databaseutils.DbConnProps dbConn)
                    throws java.sql.SQLException
```

:   Constructor 1: initialises the table (uses given database connection file)

    **Throws:**: `java.sql.SQLException`


**Method Detail**

### createArffFileTable

```
public void createArffFileTable()
                         throws java.sql.SQLException
```

:   Creates the table for the ARFF file records

    :   **Throws:**: `java.sql.SQLException`

---


### main

```
public static void main(java.lang.String[] args)
                 throws java.sql.SQLException
```

:   Main for testing

    :   **Parameters:**: `args` - **Throws:**: `java.sql.SQLException`


---


|  |  |  |
| --- | --- | --- |
| **Package** \| **Class** \| **Use** \| **Tree** \| **Deprecated** \| **Index** \| **Help** | |  |
| PREV CLASS   **NEXT CLASS** | **FRAMES**    **NO FRAMES**     **All Classes** |  |
| SUMMARY: NESTED \| FIELD \| CONSTR \| METHOD | DETAIL: FIELD \| CONSTR \| METHOD |  |


---
